# Prediction of CAF-related genes in immunotherapy and drug sensitivity in hepatocellular carcinoma: a multi-database analysis

**DOI:** 10.1038/s41435-024-00252-z

**Published:** 2024-01-17

**Authors:** Yi Yao, KaiQing Yang, Qiang Wang, Zeming Zhu, Sheng Li, Bin Li, Bin Feng, Caixi Tang

**Affiliations:** 1 Division 1, Department of Hepatobiliary and Pancreatic Surgery, Zhuzhou Central Hospital, Zhuzhou, Hunan China; 2 Division 2, Department of Hepatobiliary and Pancreatic Surgery, Zhuzhou Central Hospital, Zhuzhou, Hunan China; 3 Department of Hepatobiliary and Pancreatic Surgery, Zhuzhou Central Hospital, Zhuzhou, Hunan China

**Keywords:** Cancer genetics, Cell death and immune response

## Abstract

This study aims to identify the cancer-associated fibroblasts (CAF)-related genes that can affect immunotherapy and drug sensitivity in hepatocellular carcinoma (HCC). Expression data and survival data associated with HCC were obtained in The Cancer Genome Atlas (TCGA) and Gene Expression Omnibus (GEO) databases. Weighted correlation network analysis (WGCNA) analysis was performed to obtain CAF-related genes. Least Absolute Shrinkage and Selection Operator (LASSO) regression was used for regression analysis and risk models. Subsequently, Gene Set Enrichment Analysis (GSEA) analysis, Gene Set Enrichment Analysis (ssGSEA) analysis, Tumor Immune Dysfunction and Exclusion (TIDE) analysis and drug sensitivity analysis were performed on the risk models. Survival analysis of CAF scores showed that the survival rate was lower in samples with high CAF scores than those with low scores. However, this difference was not significant, suggesting CAF may not directly influence the prognosis of HCC patients. Further screening of CAF-related genes yielded 33 CAF-related genes. Seven risk models constructed based on CDR2L, SPRED1, PFKP, ENG, KLF2, FSCN1 and VCAN, showed significant differences in immunotherapy and partial drug sensitivity in HCC. Seven CAF-related genes may have important roles in immunotherapy, drug sensitivity and prognostic survival in HCC patients.

## Introduction

Currently, hepatocellular carcinoma (HCC) is considered one of the most common and deadly malignancies in the world, with limited treatment options available because of the complications in disease progression and therapeutic response caused by heterogeneity within the liver tumor or between each tumor case [1]. Intrahepatic or systemic metastases are the leading cause of poor prognosis in patients with advanced HCC [2], among which lung metastasis is the most common and one of the leading causes of death in HCC patients [3, 4]. Previous data shows that cirrhosis or liver fibrosis occurs in approximately 80~90% of HCC progression and is a necessary intermediate state to develop HCC. About one-third of patients with cirrhosis carry the risk of HCC through their lifetime [5, 6]. Therefore, further understanding the impact of fibroblasts in HCC and exploring the genes associated with cancer-associated fibroblasts (CAFs) in HCC may provide new ideas for therapeutic strategies for this disease.

CAFs, originating mainly from activated hepatic stellate cells and increasingly accumulating in HCC stroma, are significant sources of extracellular matrix (ECM) [7–11]. The growing evidence supported the role of CAF in tumor infiltration, migration and invasion [12, 13]. In the past decades, many CAF-related genes, such as CCL2 and CCL26, have been suggested to be involved in tumor development [14, 15]. CXCL11 in CAFs was reported for its implication in HCC migration [16]. HCC-related CAFs and their related genes may be essential in regulating HCC cells [7, 17–19]. Studies on CAFs have provided new directions for further insights into tumor development. Despite the evidence supporting the regulatory role of CAFs in HCC, research focusing on CAF-related genes in HCC remains scarce.

Weighted gene co-expression network analysis (WGCNA) is a systematic bioinformatics algorithm that integrates highly coordinated genes into multiple gene modules and analyzes the correlation between modules and target phenotypes [20]. In this study, CAF levels in HCC samples were obtained by scoring CAF to identify gene modules associated with CAF scoring. Through integrating the data in multiple databases, new CAF-related genes associated with HCC were located to analyze their effect on immunotherapy and drug sensitivity to provide a theoretical basis for further insight into the mechanisms of CAF-related genes in HCC, as well as a new direction for exploring new therapeutic tools for HCC.

## Materials and methods

### Data download and processing

Liver Hepatocellular Carcinoma (LIHC) samples in The Cancer Genome Atlas (TCGA) (https://portal.gdc.cancer.gov/) were selected and downloaded to obtain HCC gene expression data and clinical data, in which 371 HCC samples and corresponding clinical data were extracted. Microarray GSE76427 with the sequencing platform GPL10558 in GEO (https://www.ncbi.nlm.nih.gov/geo/) database was selected, which includes 115 HCC samples and corresponding clinical expression files.

### CAF scores and survival analysis

The expression data was normalized using the R language “limma” package v3.54.2. TCGA data were processed using the “averesps” function and GSE76427 microarray was processed using the “normalizeBetweenArrays” function [21]. The R (v4.2.3) “MCPcounter” package v1.2.0 was used to calculate CAF scores for data in TCGA data and GSE76427 microarray, using the “MCPcounter.probesets” and “MCPcounter.probesets” [22, 23]. The CAF scores of TCGA and GEO were merged with the clinical survival data and the survival rate was analyzed using the “survival” v3.5-3 and “surviminer” v0.4.9 packages. The surv_cutpoint function in the surviminer package was used to calculate the best cutoff value and to plot survival curves.

### WGCNA analysis

The CAF scores of TCGA and GSE76427 were extracted separately for analysis using WGCNA package v1.72-1 [20] to identify CAF-related module genes. This method analyzes the association between genes and classifies genes into different colored modules. Genes not associated with other genes are organized into insignificant gray modules. The recommended soft threshold values automatically calculated were used for the soft threshold selection procedure. The similarity between modules was computed using the R language cor function based on the eigengenes of the modules. The genes with high similarity were merged and the merged modules were analyzed to determine the correlation between module and CAFs.

### Functional enrichment analysis

Gene Ontology (GO) and Kyoto Encyclopedia of Genes and Genomes (KEGG) functional enrichment analyses were performed on the intersecting genes of the CAF-related modules from TCGA and GSE76427 analyses using R language “clusterProfiler” package v4.6.2 [24, 25]. For GO enrichment analysis, enrichGO function was performed using *p* < 0.05 as the significant pathway filter condition, while enrichKEGG function was used for KEGG pathway enrichment analysis.

### Construction or risk models

The expression data of those intersecting genes in TCGA were extracted and merged with corresponding clinical data in TCGA using for univariate cox analysis using the “survival” and “surviminer” packages, and the cxoph function to obtain the candidate genes that significantly affected the prognosis of HCC patients. The “glmnet” v4.1-7 and “surviviner” package [26, 27], as well as glmnet and cv.glmnet function, were used for LASSO regression analysis based on the candidate genes. The optimal prognostic genes (*p* < 0.05) were located based on multi-factor cox regression analysis. The point with the lowest cross-validation error was selected for feature construction using the coef function to calculate the risk score (risk score = ∑(coefficient_i_×expression of signature gene_i_)). Based on this score, risk values were assigned to each HCC sample to construct a risk model. The median risk value was used as the median value to classify the samples into high- and low-risk samples. Survival analysis was then performed on the high- and low-risk samples using the “survival” package. The calibration curves for survival period of respective 1 year, 3 year and 5 years were drawn using “survival”, “regplot” v1.1 and “rms” v6.7-1 packages, with coxph used as the function. The generated candidate genes were subjected to differential analysis using “limma” v3.54.2 and “ggpubr” v0.6.0 packages, and “wilcox.test”.

### GSEA and ssGSEA analysis

The GSEA pathway enrichment analysis was performed using the “limma” package and the “clusterProfiler” package for the high- and low-risk samples and the configuration files of “c2.cp.kegg.Hs.symbols.gmt” downloaded from GSEA database, during which the GSEA function was used. The “limma”, “GSEABase” v1.60.0 and “GSVA” v1.46.0 packages [28, 29] were used for enrichment analysis for the high- and low-risk samples. The exact configuration file “c2.cp.kegg.Hs.symbols.gmt” was used for the ssGSEA enrichment analysis. The ssGSEA scores of the relevant pathways in the samples were obtained using the gsva function and adjusted using the “normalize” function. The correlation between the risk scores of the sample and the ssGSEA scores of the signaling pathways in the risk model was carried out using the “spearman” method.

### Cancer cell line encyclopedia (CCLE) analysis

Sample expression files were downloaded from the CCLE database (https://sites.broadinstitute.org/ccle). The database includes expression files for both HCC samples and fibroblast samples. The expression data of the candidate genes in the fibroblast and HCC samples were extracted from the database and the expressions of candidate genes in fibroblast and HCC samples were analyzed using “limma” and “ggpubr” packages. The differential analysis was performed using “limma”, “ggpubr” packages and comparisons function, with the default method for analysis and *p*-value correction.

### Tumor Immune Dysfunction and Exclusion (TIDE) prediction

The TIDE database (http://tide.dfci.harvard.edu/) was used to score HCC samples from TCGA to obtain the immunotherapy score for each sample, with the higher score for better immune escape ability and worse immunotherapy efficiency. During the analysis, the tumor type was selected as other due to the absence of a specific HCC category in the database, and the previous immunotherapy type was chosen as no. The file data were uploaded using the HCC expression data downloaded from TCGA database, and the data were log2 processed to meet the data type required by the database. Subsequently, the differences in immunotherapy scores between the high- and low-risk samples were analyzed using the “wilcox.test” method. The analysis of differences in immunotherapy response in the high- and low-risk samples was carried out using “Chi-squared Test “.

### Drug sensitivity analysis

The Genomics of Drug Sensitivity in Cancer (GDSC) database (https://www.cancerrxgene.org/) was used to download tumor expression data and drug data, based on which “oncoPredict” v0.2 package was used to analyze drug sensitivity [30]. The GDSC data were used as the train group and the risk model data as the test group, and the batch correction method was “eb “. A fluctuation deletion threshold of 0.2 and a minimum sample size of 10 were used to obtain the drug sensitivity analysis results and the sensitivity scores for each drug for each sample. Subsequently, “limma” and “ggpubr” package were used to analyze the differences in drug sensitivity between the high- and low- risk samples in the risk model using “wilcox. test”.

## Results

### CAF scoring and survival analysis in HCC

**Fig. 1:**
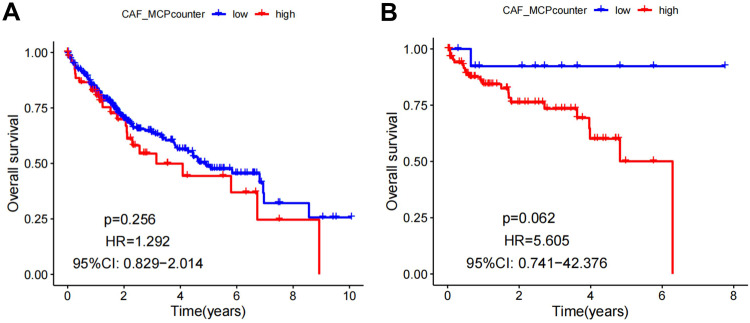
Overall survival based on CAF scoring in TCGA and GSE databases using MCPcounter. **A** Survival rate in TCGA database; **B** Survival rate in GEO data, red lines in the graph are high CAF scoring and blue are low CAF scoring.

CAF scoring was performed on HCC expression data using the MCPcounter algorithm to obtain a fibroblast score for each tumor (Supplementary Tables 1 and 2). Subsequently, survival analysis was performed based on the CAF scoring. The results showed that among HCC data in TCGA, the survival rate of high CAF-scoring samples was lower than that of the low CAF-scoring samples, but this comparison showed no significant difference (Fig. 1A). Meanwhile, survival analysis of the CAF scoring of the tumor samples from GSE76427 downloaded from the GEO database also showed that the survival rate of the high CAF scoring samples was lower than that of the low CAF scoring samples (Fig. 1B), although no statistically significant difference was detected. Those results imply that CAFs may influence the survival of HCC patients indirectly.

### WGCNA analysis

**Fig. 2:**
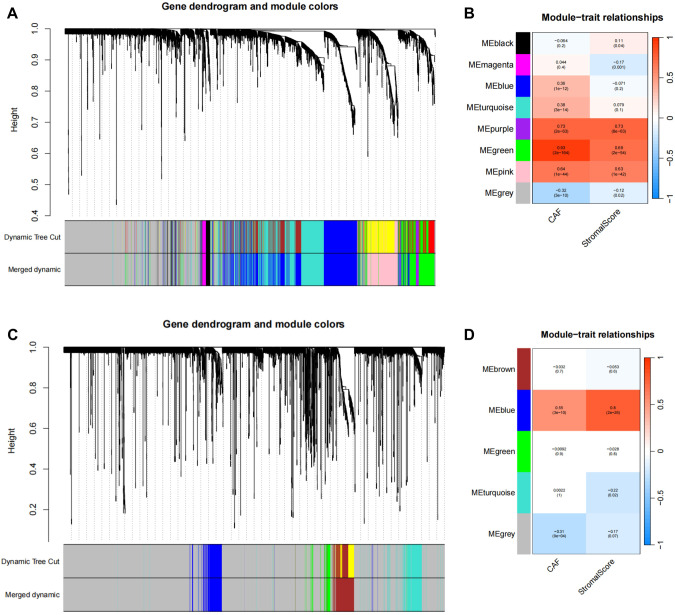
WGCNA analyses of CAF-related genes. **A** WGCNA analysis of TCGA data, graph A shows gene module clustering, the upper part shows clustering before module merging, the lower part shows clustering after module clustering; **B** Correlation analysis between module and CAF scoring, the horizontal coordinates indicate CAF scoring and StromalScore respectively, the vertical coordinates indicate gene modules, the right histogram is the color scale; **C, D** WGCNA analysis of the GEO database.

To further screen HCC-associated CAFs, WGCNA analysis was performed on TCGA data and GSE76427 data. WGCNA analysis of TCGA data showed that all genes were classified into 8 different colored modules (Fig. 2A). As CAFs were usually expressed in the cell stroma, these modules were analyzed for correlation with CAF scoring and StromalScore (Fig. 2B) and the results revealed that the green module was highly correlated with CAF and StromalScore, implying that genes in this module may be closely associated with HCC associated CAFs. Similarly, WGCNA analysis of GSE76427 revealed that the genes in this chip were divided into 5 different colored modules (Fig. 2C), and correlation analysis showed a significant positive correlation between the blue module and CAF and StromalScore (Fig. 2D), implying that the genes in the blue module may be closely associated with CAFs.

### Functional enrichment on candidate genes

**Fig. 3:**
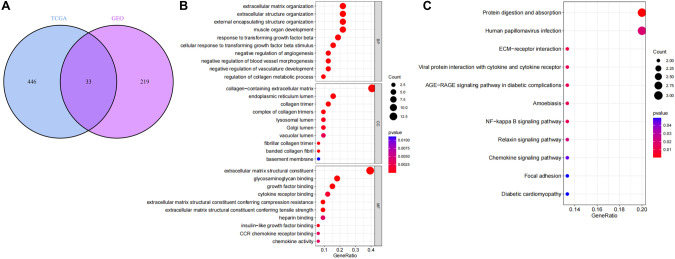
Screening and functional enrichment of CAF candidate genes. **A** Gene intersections in modules significantly associated with CAFs of TCGA and GEO, with intersecting genes in the middle part; **B** GO functional enrichment analysis of intersecting genes, with horizontal coordinates indicating GeneRatio, vertical coordinates indicating functional entries, and color scale in the right histogram; **C** KEGG pathway of intersecting genes enrichment analysis.

The genes in the significantly correlated modules of TCGA and GSE76427 were extracted and the two sets of gene data were intersected (Fig. 3A), and 33 candidate genes were finally obtained. These 33 candidate genes were further analyzed for GO and KEGG functional enrichment, and were found to be enriched mainly under the functional categories of “extracellular matrix organization” and “protein digestion and absorption” (Fig. 3B, C), indicating those functional categories may be associated with the formation or changes of CAFs in HCC. In the signaling pathway analysis, we found that these genes were also significantly enriched in the “AGE-RAGE signaling pathway in diabetic complications” and “NF-kappa B signaling pathway”. Those results suggest that alternation in CAF levels may influence these pathways and thus affect the development of HCC.

### One-way cox analysis of candidate genes

**Fig. 4:**
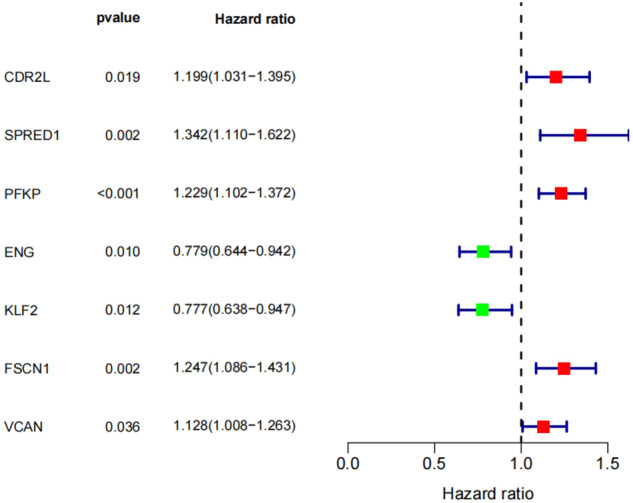
One-way cox analysis of candidate genes, which shows genes significantly associated with survival rate of HCC patients after one-way cox analysis.

One-way cox analysis was performed on these 33 candidate genes (Fig. 4, Supplementary Table 3), and the results revealed that 7 genes, including CDR2L, were significantly associated with prognostic survival of HCC patients, suggesting these 7 genes may be related to CAFs in HCC and may play an important role in the prognosis of HCC patients.

### LASSO regression risk models

**Fig. 5:**
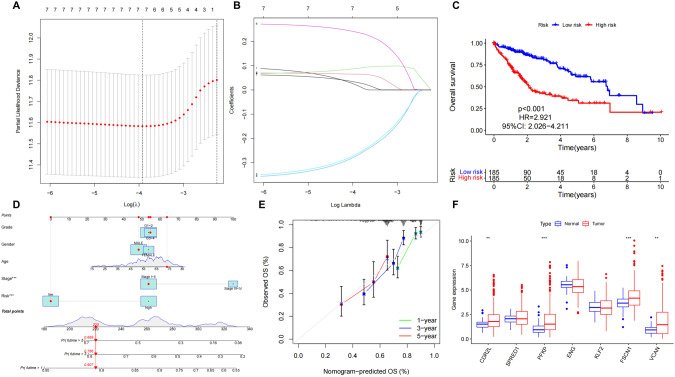
LASOO regression analysis and risk model constructions of the 7 candidate genes. **A**, **B** LASOO regression of risk genes obtained from one-way cox analysis; **C** Survival analysis of risk model constructed from LASSO regression, red lines in the graph are the high risk samples and blue lines are the low risk samples. **D** Line chart for risk model; **E** Calibration curves for survival period of respective 1 year, 3 year and 5 years; **F** Differential expression of candidate genes, red box in the graph is HCC samples and blue box is normal sample (*N* = 50, *T* = 374) (*p.val < 0.05, **p.val < 0.01, ***p.val < 0.001).

The LASSO regression risk model was constructed based on the 7 genes, CDR2L, SPRED1, PFKP, ENG, KLF2, FSCN1 and VCAN. Further survival analysis of the risk model constructed by LASSO regression (Fig. 5C) revealed that the survival rate in the high-risk samples was significantly lower than that of the low-risk samples, indicating that the 7 genes were not only closely associated with CAFs but also played important roles in the prognosis of HCC patients. Based on the data in the line chart and calibration curves (Fig. 5D, E), there were significant difference in patient risk and stage, which indicates this risk model is of high accuracy to predicting the prognosis of patients. Then the 7 candidate genes were analyzed for differential expression in HCC and normal samples (Fig. 5F), which identified CDR2L, PFKP, FSCN1 and VCAN were highly expressed in HCC samples, whereas the expressions of SPRED1, ENG and KLF2 in HCC samples showed no significant difference with that in normal samples. Those results suggested CDR2L, PFKP, FSCN1 and VCAN may regulate HCC more directly, while SPRED1, ENG and KLF2 may mediate HCC progression indirectly.

### Expression analysis of model genes and CAF marker genes

**Fig. 6:**
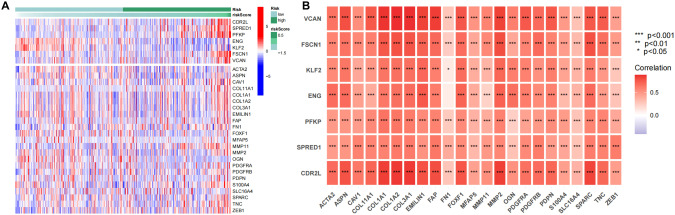
Expressions of model genes and CAF marker genes. **A** The expressions of genes used to construct the risk model and the expression of CAF marker genes in the risk model, the vertical coordinates in the graph indicate the gene names, the top on the heat map is the model gene, the bottom of the heat map is the CAF marker genes, and the right histogram is the color scale; **B** Correlation analysis between model genes and CAF marker genes, the horizontal coordinates in the graph indicate the CAF marker gene, the vertical coordinate indicates the model gene, and the right histogram is the color scale.

CAF marker genes were obtained from published literature [31, 32]. Subsequently, the expression of genes screened for modeling as well as CAF marker genes in the risk model were further analyzed and the heat map was plotted (Fig. 6A). The results showed that most of the CAF marker genes had higher expression levels in the high-risk samples, while the model genes CDR2L, SPRED1, PFKP, FSCN1 and VCAN also had higher expression levels in the high-risk samples, which was consistent with the above results. The positive correlation between the above 7 model genes and CAF marker genes was found (Fig. 6B), suggesting that the expressions of these 7 genes might be closely related to CAF levels in HCC.

### Risk model GSEA analysis

**Fig. 7:**
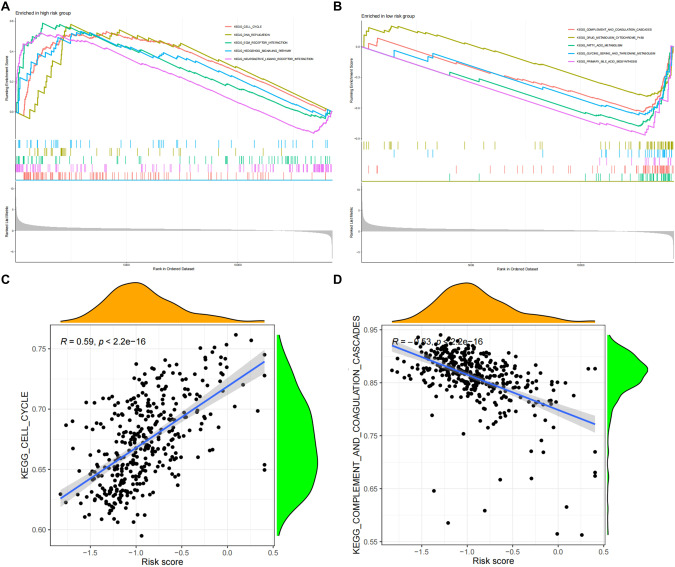
GESA analysis of the risk model. **A**, **B** GSEA enrichment for part of significant pathways in the high- and low-risk samples, respectively; **C**, **D** Correlation analysis between the enriched pathway scores obtained from GSEA pathway enrichment and the risk values in the risk model.

To further understand the functions of genes in the risk model, GSEA pathway enrichment analysis was performed on high- and low-risk samples (Fig. 7A, B). The results showed that signaling pathways, such as CELL CYCLE, DNA REPLICATION were significantly enriched in the high-risk samples, while COMPLEMENT AND COAGULATION CASCADES, DRUG METABOLISM CYTOCHROME P450 were significantly enriched in the low-risk samples. These pathways may play an important regulatory function in HCC. Meanwhile, ssGSEA analysis was performed on the risk model and the risk values of each sample were calculated for correlation analysis with the pathways obtained from ssGSEA analysis (Fig. 7C, D, Supplementary Figs. 1–6). The results found a significant correlation between the risk scores of the samples and the signaling pathways such as CELL CYCLE, which implies that CAF most likely regulates HCC progression by indirectly mediating the activation of these pathways.

### CCLE expression analysis

**Fig. 8:**
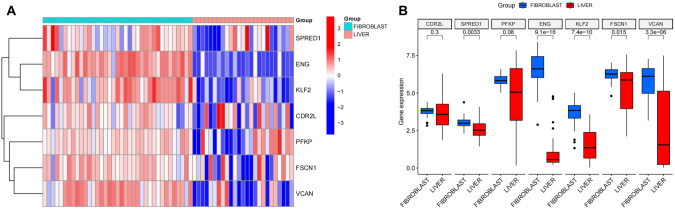
Candidate gene expression analysis in CCLE. **A** Heat map of candidate gene expression in CAFs and HCC samples in CCLE, with color scale on the right histogram; **B** Box plot of candidate gene expression in CAFs and HCC in CCLE.

To further understand the expression of the 7 candidate genes in HCC samples and CAF samples, the differential expressions of CDR2L, SPRED1, PFKP, ENG, KLF2, FSCN1 and VCAN were analyzed using CCLE database (Fig. 8A, B). The results showed that expression levels of these 7 genes were all down-regulated in the HCC samples compared to CAF cells, among which 6 genes, SPRED1, PFKP, ENG, KLF2, FSCN1 and VCAN, were significantly down-regulated in HCC. Those results further suggest that these 7 genes are closely related to CAF levels in HCC, and their expression levels may be substantially associated with CAF. Therefore, it is speculated that CAFs regulate HCC progression most likely through regulating the expressions of these 7 genes.

### Risk model immunotherapy

**Fig. 9:**
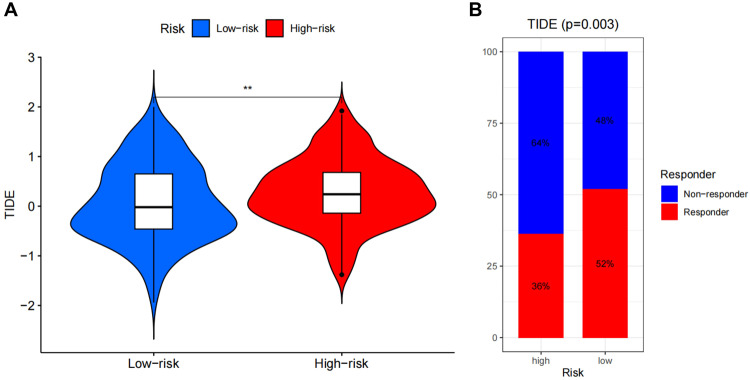
TIDE analysis on risk models. **A** Differential analysis in TIDE scores in the risk model, horizontal coordinates indicate TIDE scores, vertical coordinates indicate risk model groupings, the higher the TIDE score, the greater the immune escape capacity of that sample; **B** Proportion of samples responding to immunotherapy in the risk model.

To further understand the sensitivity of the risk model to immunotherapy, the samples in TCGA database were scored using TIDE scoring to obtain the immunotherapy scoring (Supplementary Fig. 7 and Supplementary Table 4). Subsequently, a differential analysis of immunotherapy scoring between the high- and low-risk samples in the risk model (Fig. 9A) was performed and showed that the TIDE scores were significantly higher in the high-risk samples than those in the low-risk samples, indicating that the high-risk samples had a higher potential for immune escape and weaker immunotherapy efficiency. The proportion of samples responding to immunotherapy in the high- and low-risk samples was counted (Fig. 9B) and the proportion of non-responders was higher in the high-risk samples than in the responders. In contrast, the opposite expression pattern was found in the low-risk samples. This result suggests that these 7 CAF-related genes, which were used to construct the risk model, may be associated with immunotherapy for HCC, highlighting the potential of CAF on immunotherapy in HCC.

### Drug sensitivity prediction on risk models

**Fig. 10:**
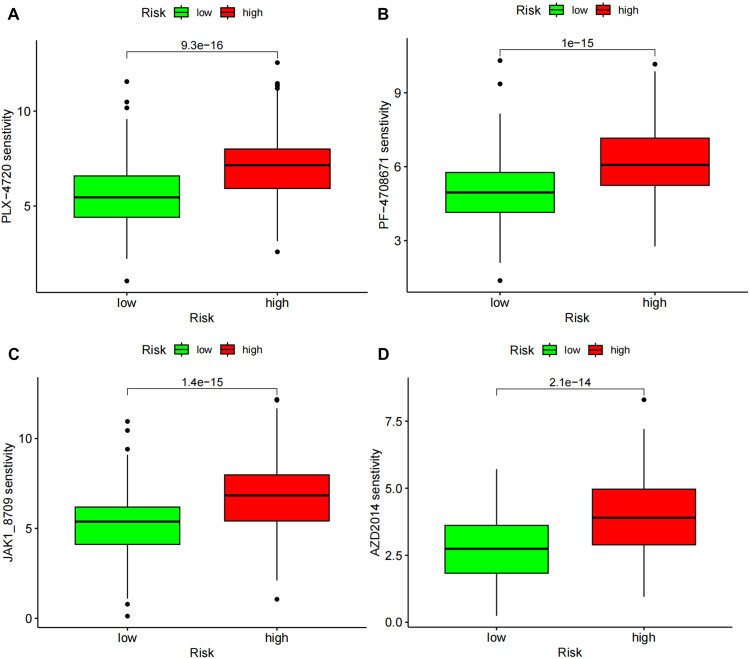
Drug sensitivity analysis using risk models. **A–D**: Sensitivity analysis of 4 different drugs in the risk model. Vertical coordinates in the graph indicate drug sensitivity, horizontal coordinates indicate sample subgroups, and *p*-values for differences are shown above.

To further screen the drugs for HCC, drug sensitivities in the risk model were predicted and differences in drug sensitivities between the high- and low-risk samples were analyzed. The results showed that 134 drugs showed significant differences in sensitivity between the high- and low-risk samples (Supplementary Table 5), among which PLX-4720, PF-4708671, JAK1_8709 and AZD2014, had the highest statistical differences (Fig. 10A–D), implying that these drugs had better therapeutic effects in the CAF-related gene constructed HCC risk model. Additionally, drug sensitivity analysis demonstrated that all four drugs show better sensitivity in the high-risk samples, suggesting that these 4 drugs may be more effective in treating CAF-associated HCC.

## Discussion

CAFs were reported for their regulation of tumor growth, angiogenesis and metastatic behavior in HCC [16, 33–35]. CAFs can promote metastasis in HCC through chemokines such as CXCL11, and CAF-carried proteins, such as CCL2 and CCL7 have been shown to enhance HCC progression [13, 36–38]. The clinical analysis found CAF abundance is often associated with poor clinical outcomes and is considered an attractive therapeutic target in primary HCC [39]. The bioinformatics method provides clues in predicting CAF-related genes and prognosis in HCC. Dong et al. found that TOP2A might be closely related to CAF in HCC cancer and affect the prognosis of patients with HCC [40]. Song et al. predicted the prognostic value of CAF in HCC using XCELL calculation and found that the CAF-related risk model constructed based on genes such as LAMB1 is of great potential in predicting the prognosis of patients with HCC [41]. In addition, Yu et al. constructed a CAF-associated risk model in HCC for predicting the prognosis based on single-cell sequencing data and bulk RNA-seq data [42]. Nevertheless, there are few studies identifying the CAF-related genes in HCC, and there is still a paucity of studies exploring the relevant mechanisms of CAF in HCC prognosis. In this study, we obtained CAF scores from HCC data and GSE76427 in TCGA by using the MCPcounter algorithm and found that HCC patients with higher CAF scores had worse survival rates based on the data from TCGA data and the GSE76427. However, CAF scores were not significantly associated with prognostic survival of HCC patients, but in other tumors, such as gastric cancer, CAF abundance was found to be strongly associated with prognosis [43, 44]. This inconsistency may be explained by data limitations, or on the one hand, and suggests that CAF may not directly and dramatically affect the survival rate in HCC patients but in an indirect way.

To understand the regulatory mechanisms associated with CAFs in HCC, CAF-related genes in TCGA and GSE76427 data were predicted by the WGCNA method, which is the first time to search for CAF-related genes in HCC by the WGCNA analysis. A total of 33 shared CAF-associated genes were predicted in the two datasets. Signaling enrichment analysis of these associated genes showed that they were enriched in the “AGE-RAGE signaling pathway in diabetic complications” and the “NF-kappa B signaling pathway”. Consistently, several studies showed that these signaling pathways are significantly involved in the development of HCC [45–49], which further confirms that CAFs may indirectly influence the development of HCC through these CAF-related genes.

Risk models for HCC were constructed based on CDR2L, SPRED1, PFKP, ENG, KLF2, FSCN1 and VCAN genes using one-way cox analysis and LASSO regression algorithms. Further analysis of the expression of these 7 CAF-related genes showed that their expression levels in HCC samples were significantly correlated with the expression levels of CAF marker genes in HCC samples, which further proved the association of 7 CAF-related genes with CAF abundance in HCC. The decreased expression of SPRED1 can substantially influence HCC progression [50]. The study by Tan et al. showed miR-126-3p can affect the sensitivity of HCC cells to Sorafenib by regulating SPRED1 [51]. In addition, SPRED1 is thought to be a regulator in several tumors, including gastric cancer and breast cancer [52–54]. SPFKP was found to affect cell stemness of HCC cells [55], whereas in lung cancer, PFKP was found to regulate glucose metabolism [56]. In leukemia, PFKP affected disease progression by influencing CXCR4-dependent T-cell infiltration [57].

However, there are limited studies that have researched the regulatory mechanisms of PFKP in HCC. In the study of Li et al., it was pointed out that KLF2 can inhibit TGF-β-mediated cancer cell motility, thus affecting HCC development [58]. Moreover, KLF2 was reported as a promising marker in HCC and could be used as a prognostic marker associated with fibrosis and immune infiltration for advanced HCC [59]. Accumulating evidence highlighted that KLF2 may be regulated by lncRNAs such as FBXL10-AS1 and GHET1, thus affecting HCC progression [60–64], and that KLF2 can mediate HCC progression through c-myc [65]. It was also noted that miRNAs such as miR-145, miR-133a, and miR-539 could influence proliferation, migration, and invasion ability of HCC cells by targeting FSCN1 [66–69]. LncRNA ADORA2A-AS1 was found to modulate the FSCN1/AKT axis to regulate HCC progression [70]. FSCN1 influenced adriamycin resistance in HCC by modulating epithelial-mesenchymal transition [71]. In the study by Hayashi et al., FSCN1 was also found to modulate the expression of E-cadherin in HCC [72]. The implication of VCAN in HCC was reported in a previous study, which demonstrated that VCAN could affect the proliferation and metastasis of HCC cells through EGFR-PI3K-AKT pathway [73]. Additionally, VCAN protein secreted by CAFs can increase the cellular malignant transformation of HCC. Therefore VCAN was considered to have the potential to serve as a new therapeutic target in HCC [74]. Although less research reported the mechanism of CDR2L and ENG in HCC, CDR2L was found to be a hub gene in paraneoplastic cerebellar degeneration [75, 76]. CDR2L has also been suggested as a regulatory gene in ovarian cancer [77]. ENG, as an endothelial glycoprotein gene, has been known for its essential role in many tumors with the potential as a target for tumor therapy [78–80]. The expression level of CAF-related ENG was found to influence the invasion and metastasis of rectal cancer cells, as reported by Paauwe et al. [81]. Although the regulatory roles of these genes have been reported in different tumors, however, few studies reported their role in HCC, in HCC related CAFs. In the present study, HCC expression data were analyzed to search for CAF-related genes in HCC. Further expression analysis of these 7 model genes in the CCLE showed that they exhibited significantly elevated expression levels in fibroblasts. TIDE online algorithm in the risk model found that the low-risk samples were closely associated with improved immunotherapy efficiency in HCC patients. Drug sensitivity analysis demonstrated that the high-risk samples were highly sensitive to drugs such as PLX-4720, PF-4708671, JAK1_8709 and AZD2014. Those analyses also provide new research ideas for HCC treatment. In this study, several new CAF-related genes were obtained using different CAF score calculation methods and data from different sources instead of those already reported. The risk model constructed shows high predictive ability for the prognosis of patients with HCC, and therefore, provides a broader idea and direction for further understanding the CAF-related regulatory mechanisms in HCC.

Although this study provides a new direction for further understanding CAF-related genes and their regulatory mechanisms in HCC, we should still be aware of some limitations. First, this is a retrospective bioinformatics analysis based on public expression data, and thus the prognostic and therapeutic value of the model constructed based on these 7 CAF-related genes should be cross-validated in more data. In addition, the specific biological role of CAF-related genes in HCC and their association with CAFs should be further clarified through molecular and animal experiments. Nevertheless, our results can serve as a theoretical basis for future studies on CAF-related studies in HCC.

## Conclusion

In conclusion, a comprehensive co-expression network focusing on CAF-related genes in HCC was constructed. Risk models constructed based on CAF-related genes, CDR2L, SPRED1, PFKP, ENG, KLF2, FSCN1 and VCAN were able to predict their association with prognosis, chemotherapy and immunotherapy response of HCC patients. This study provides new insights into CAF-related regulation and therapeutic strategies for HCC. Clinical validation would further added the credibility of this study and future studies with clinical data are encouraged to further validate the association of CDR2L, SPRED1, PFKP, ENG, KLF2, FSCN1 and VCAN with prognosis, chemotherapy and immunotherapy response of HCC patients.

### Supplementary information

supplementary figures

supplementary table legends

Supplementary Table 1

Supplementary Table 2

Supplementary Table 3

Supplementary Table 4

Supplementary Table 5

## Data Availability

All data are uploaded as supplementary materials.
