# Supplementary material for: Prediction of CAF-related genes in immunotherapy and drug sensitivity in hepatocellular carcinoma: a multi-database analysis
Source: Genes Immun. 2024 Jan 17;25(1):55–65. doi: 10.1038/s41435-024-00252-z (PMC10873201; doi:10.1038/s41435-024-00252-z)
Supplement: Supplementary file 1 — supplementary figures [file 41435_2024_252_MOESM1_ESM.pdf]

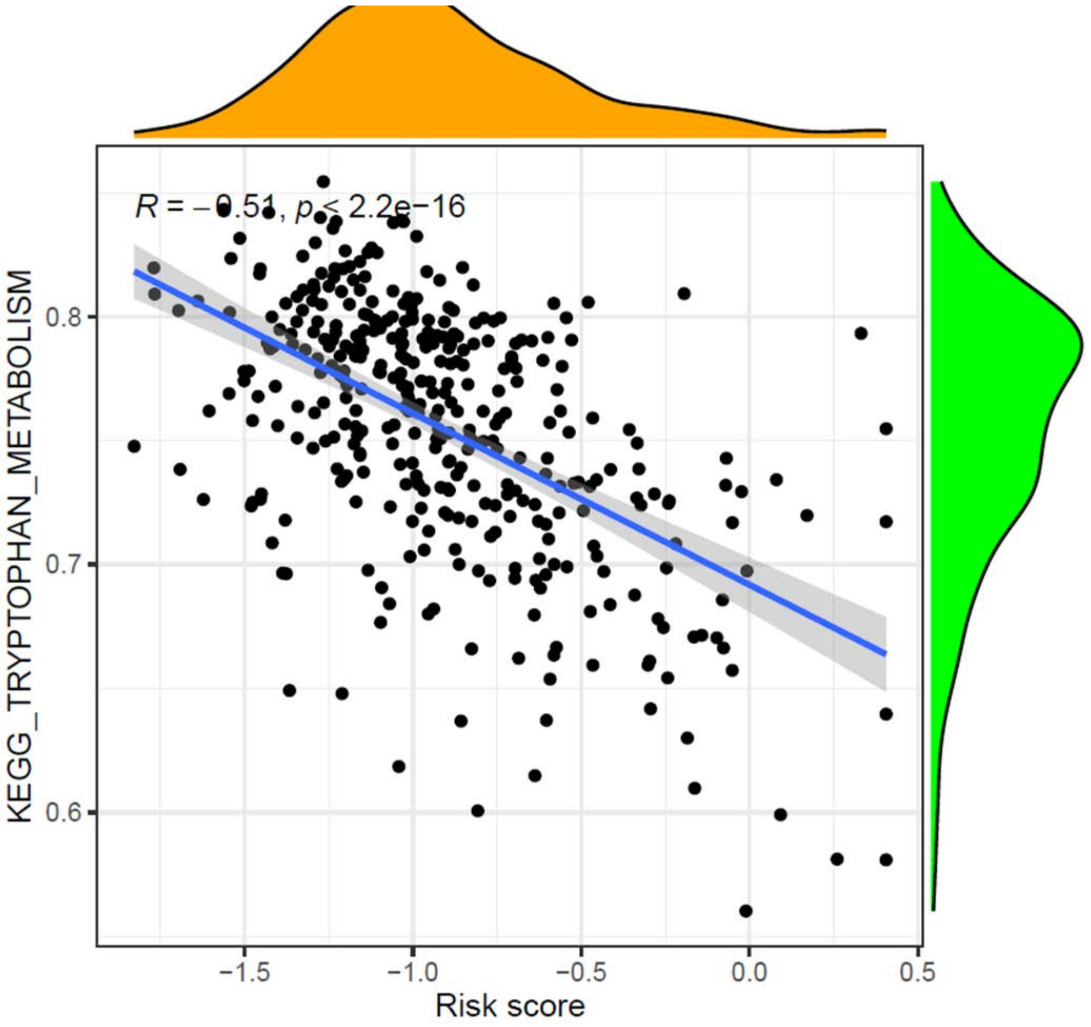

Supplementary Figure 1.ssGSEA analysis was performed on the risk model and the risk values of each sample were calculated for correlation analysis with the pathways obtained from ssGSEA analysis

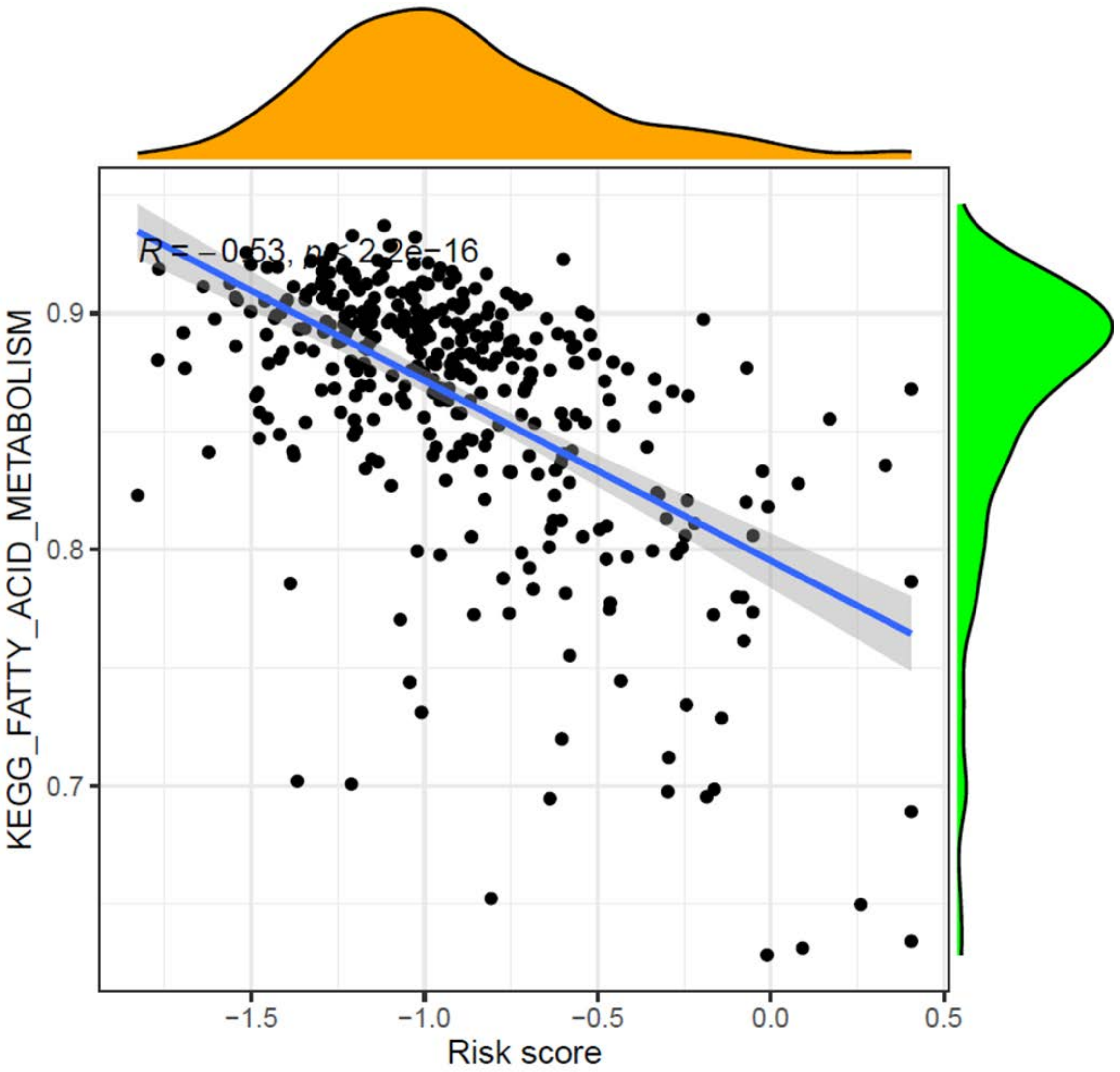

Supplementary Figure 2.ssGSEA analysis was performed on the risk model and the risk values of each sample were calculated for correlation analysis with the pathways obtained from ssGSEA analysis

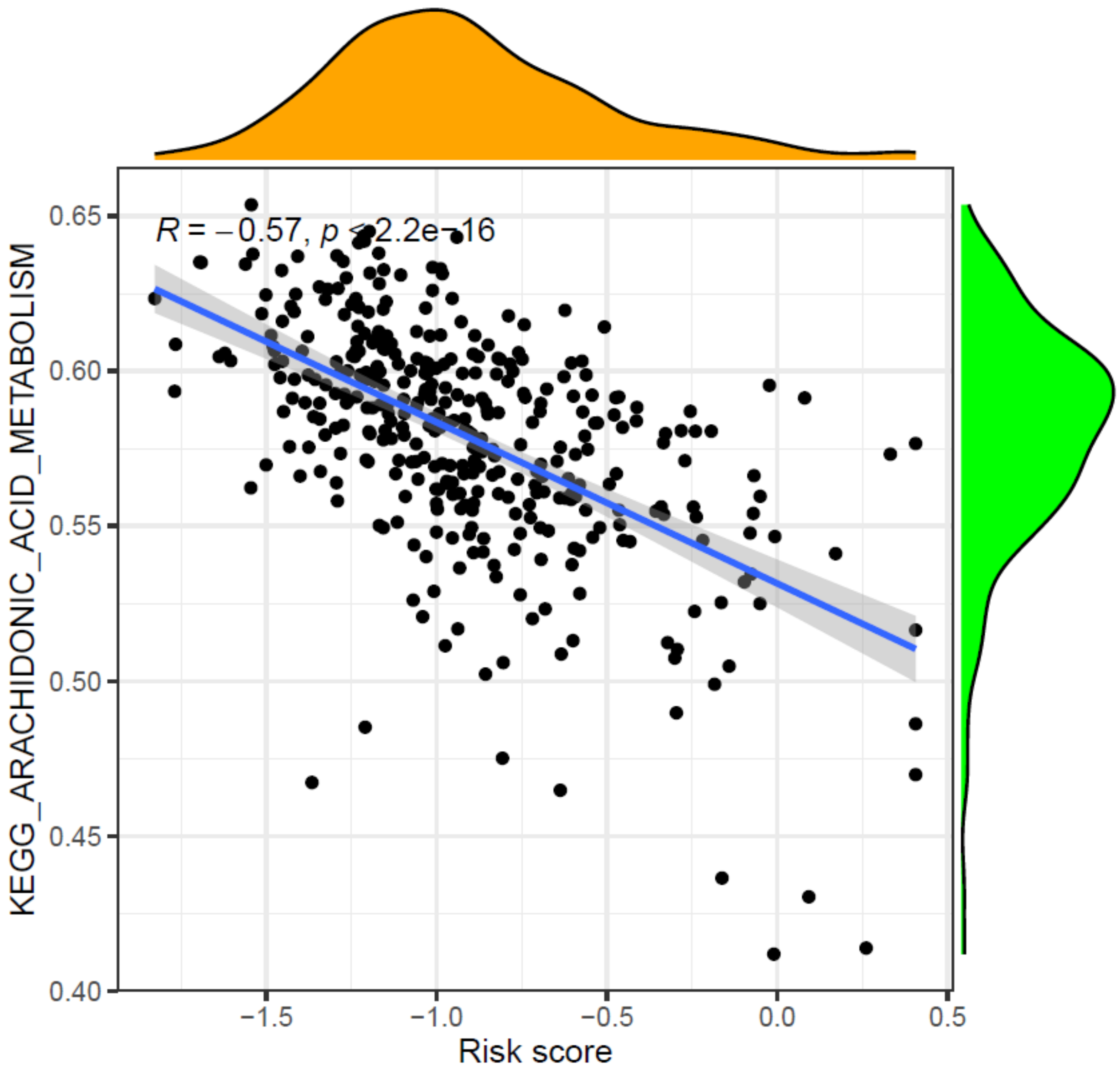

Supplementary Figure 3.ssGSEA analysis was performed on the risk model and the risk values of each sample were calculated for correlation analysis with the pathways obtained from ssGSEA analysis

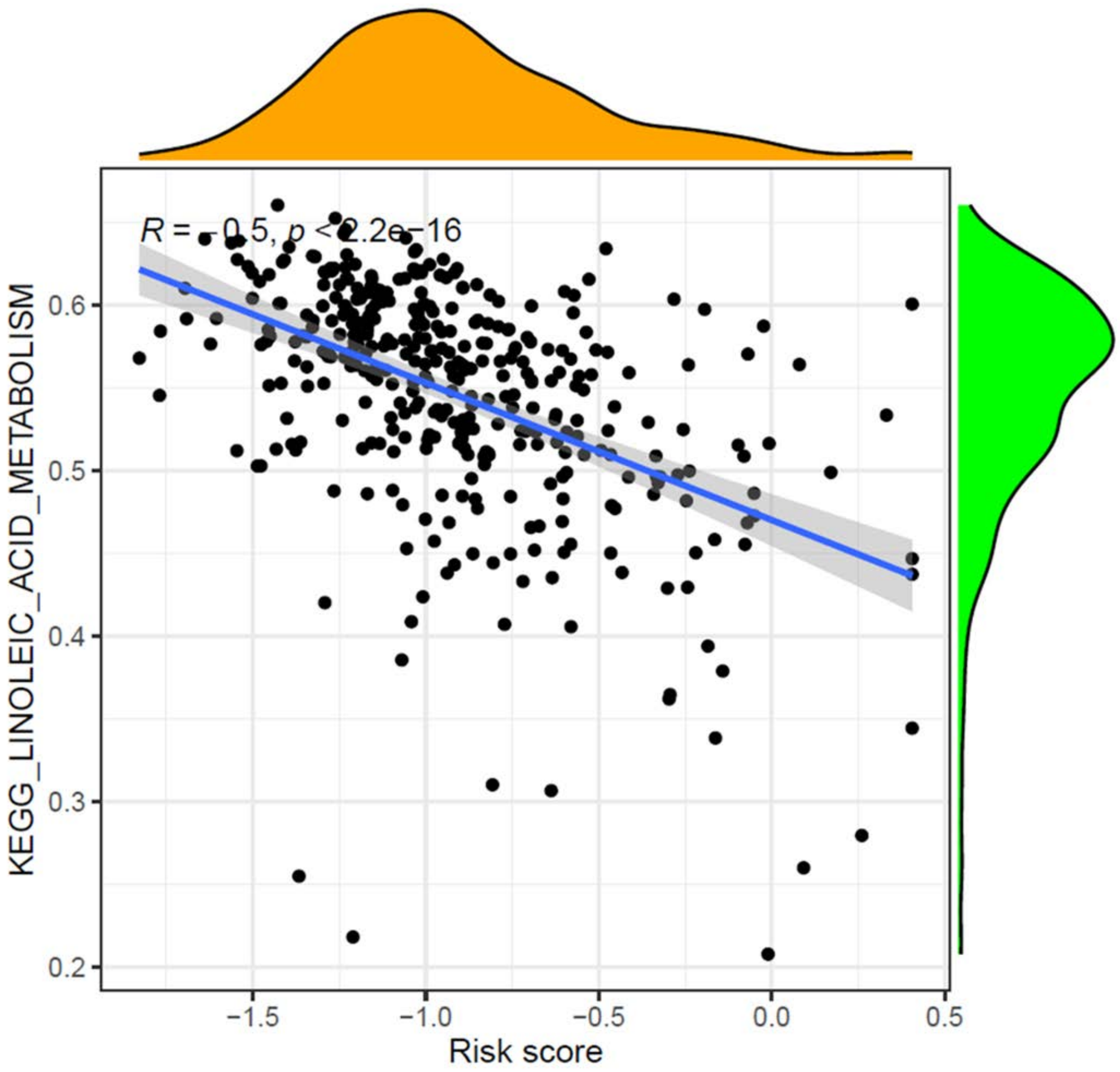

Supplementary Figure 4.ssGSEA analysis was performed on the risk model and the risk values of each sample were calculated for correlation analysis with the pathways obtained from ssGSEA analysis

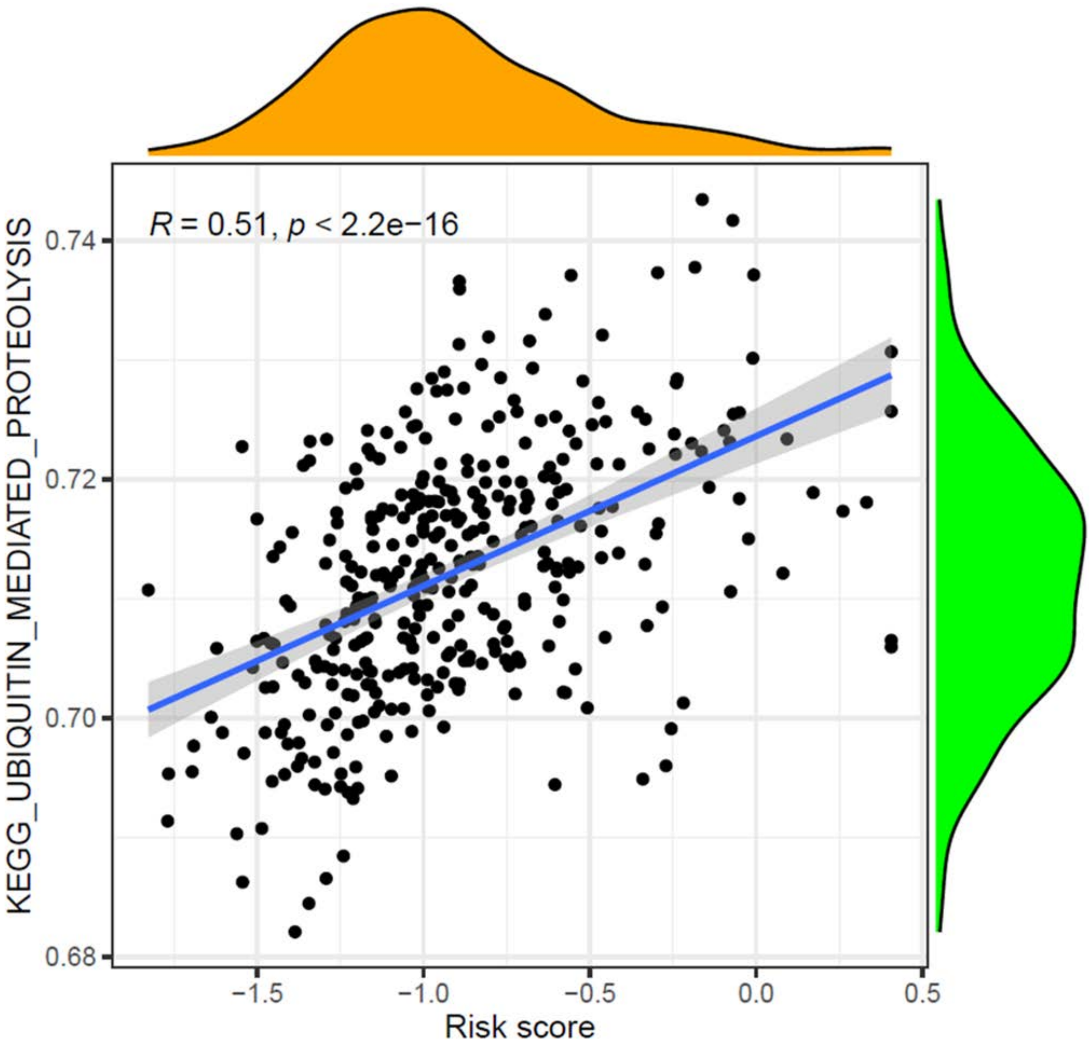

Supplementary Figure 5.ssGSEA analysis was performed on the risk model and the risk values of each sample were calculated for correlation analysis with the pathways obtained from ssGSEA analysis

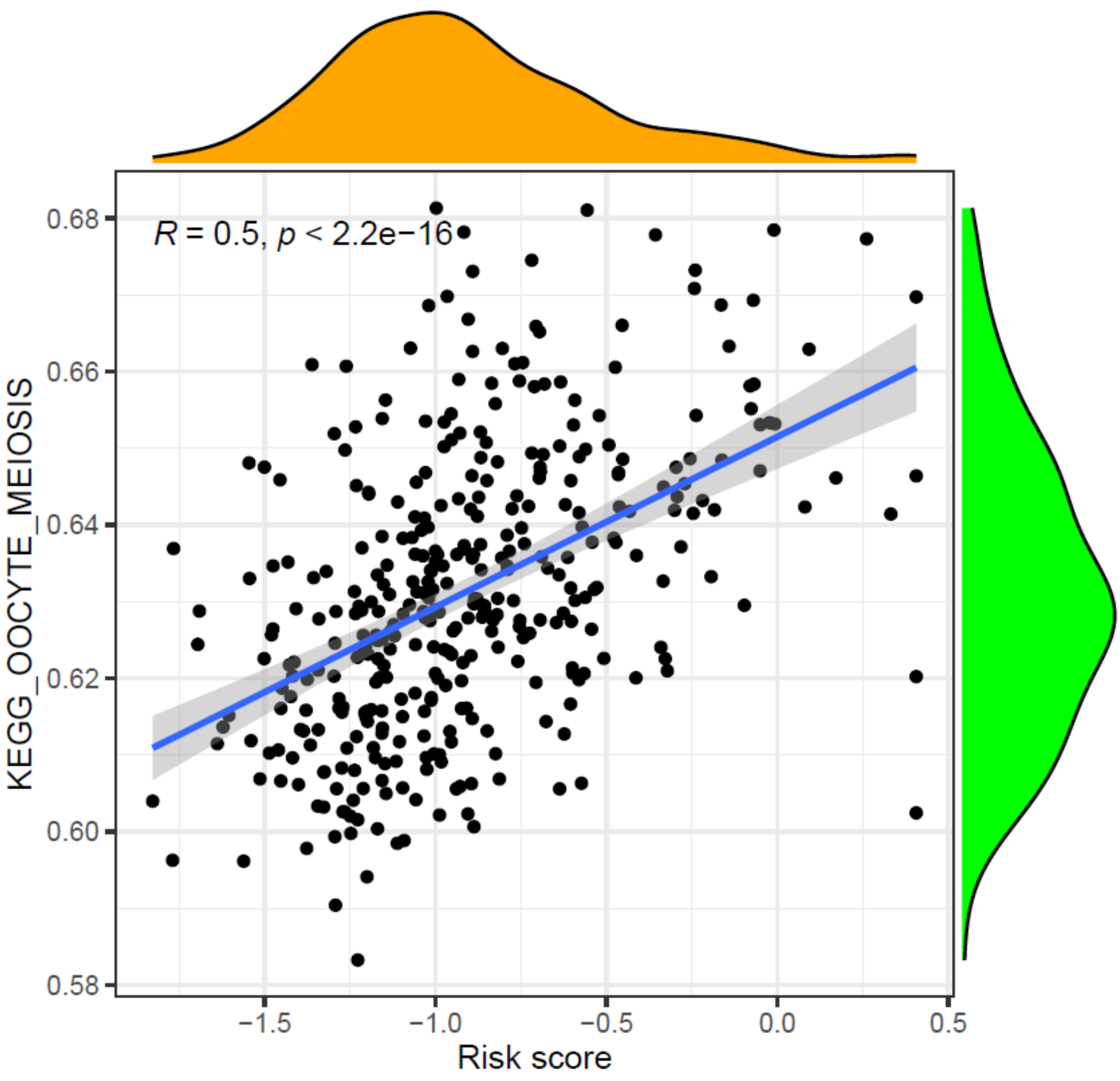

Supplementary Figure 6.ssGSEA analysis was performed on the risk model and the risk values of each sample were calculated for correlation analysis with the pathways obtained from ssGSEA analysis

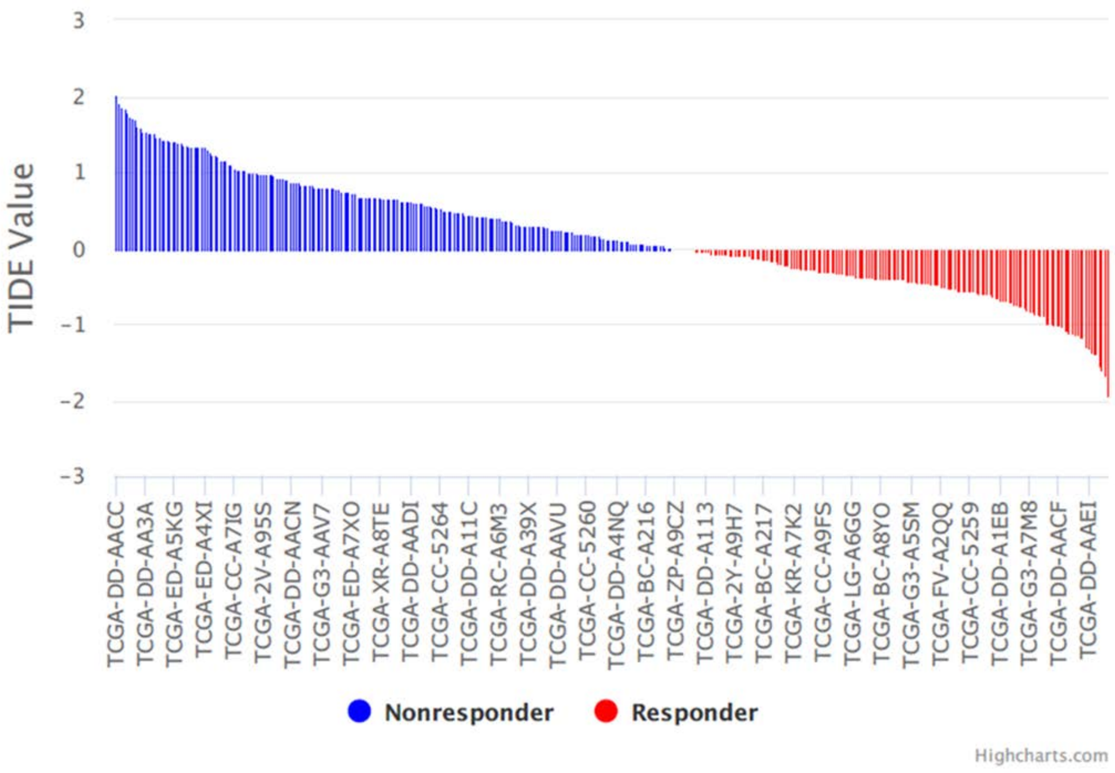

Supplementary Figure 7. Samples in TCGA database were scored using TIDE scoring to obtain the immunotherapy scoring
