# Supplementary material for: Prediction of CAF-related genes in immunotherapy and drug sensitivity in hepatocellular carcinoma: a multi-database analysis
Source: Genes Immun. 2024 Jan 17;25(1):55–65. doi: 10.1038/s41435-024-00252-z (PMC10873201; doi:10.1038/s41435-024-00252-z)
Supplement: Supplementary file 2 — supplementary table legends [file 41435_2024_252_MOESM2_ESM.doc]

Supplementary Table 1-2. CAF scoring was performed on HCC expression data using MCPcounter algorithm to obtain a fibroblast score for each tumor

Supplementary Table 3. One-way cox analysis was performed on these 33 candidate genes

Supplementary Table 4. TIDE scores for samples in TCGA database to obtain the immunotherapy scoring

Supplementary Table 5. Sensitivity differences between high-risk and low-risk samples of 192 drugs
